# Cellular and Molecular Mechanism of Traditional Chinese Medicine on Ventricular Remodeling

**DOI:** 10.3389/fcvm.2021.753095

**Published:** 2021-12-01

**Authors:** Yong-Chun Zhu, Bo Liang, Ning Gu

**Affiliations:** ^1^Nanjing University of Chinese Medicine, Nanjing, China; ^2^Nanjing Hospital of Chinese Medicine Affiliated to Nanjing University of Chinese Medicine, Nanjing, China

**Keywords:** ventricular remodeling, traditional Chinese medicine, mechanism, cardiovascular disease, review

## Abstract

Ventricular remodeling is related to the renin-angiotensin-aldosterone system, immune system, and various cytokines involved in inflammation, apoptosis, and cell signal regulation. Accumulated studies have shown that traditional Chinese medicine can significantly inhibit the process of ventricular remodeling, which may be related to the mechanism mentioned above. Here, we conducted a system overview to critically review the cellular and molecular mechanism of traditional Chinese medicine on ventricular remodeling. We mainly searched PubMed for basic research about the anti-ventricular remodeling of traditional Chinese medicine in 5 recent years, and then objectively summarized these researches. We included more than 25 kinds of Chinese herbal medicines including Qi-Li-Qian-Xin, Qi-Shen-Yi-Qi Pill, Xin-Ji-Er-Kang Formula, and Yi-Qi-Wen-Yang Decoction, and found that they can inhibit ventricular remodeling effectively through multi-components and multi-action targets, which are promoting the clinical application of traditional Chinese medicine.

## Introduction

Ventricular remodeling, referring to a variety of injuries that change the original substances and cardiac morphology of the heart, is an adaptive response of the body and a pathophysiological process of lesion repair, overall ventricular compensation, and secondary pathophysiology (1). Ventricular remodeling occurs in response to cardiac disease or cardiac damage, and the common causes are myocardial infarction, hypertension, cardiomyopathy, and valvular disease (2, 3). The histological manifestations include the enlargement of the ventricular cavity, a progressive decrease of cardiac function, extracellular collagen deposition, inflammatory cell infiltration, apoptosis, and so on (4). It is also accompanied by neurological and humoral changes, volume overload, and other pathophysiological processes (3, 5). Its mechanism is related to the neuroendocrine system [including sympathetic nervous system (SNS) and renin-angiotensin-aldosterone system (RAAS)], immune system, and various cytokines involved in inflammation, apoptosis, and cell signal regulation (6).

Traditional Chinese medicine, mainly from the East, has shown its idiographic ascendancy in the prevention, therapeutic effect, rehabilitation, and health care of diverse diseases (7, 8). The evidence-based use of traditional Chinese medicine (TCM) keeps a foothold in China and other Asian countries, and with the popularity of TCM in the East, it is increasingly accepted and used by other countries globally (9, 10). Accumulated evidence indicates that TCM has a better effect on ventricular remodeling (VR) compared with western medicine with a single active ingredient, and the relevant mechanism is being carried out. However, we still cannot fully understand the anti-VR mechanism of TCM. Here, we shed light on recent advances in therapeutic VR of TCM and try to list the main anti-VR mechanisms of TCM. We further summarized preclinical findings and described some current problems and challenges to provide a prospect in this field.

## Molecular Pathways Involved in Cardiac Remodeling

The pathogenesis of VR is associated with several molecular pathways and their relative importance depends on the underlying cause of VR. Inflammatory signals seem to be more important in VR, which are associated with the intense activation of cytokine cascades (11–13). Furthermore, oxidative stress, apoptosis, and autophagy, transforming growth factor β1 (TGF-β1), neuroendocrine system, and peroxisome proliferators-activated receptor γ (PPARγ) are involved in VR regardless of etiology (13, 14).

### Inflammatory Cytokines

In recent years, a large number of studies have confirmed that the immune response triggered by a myocardial injury, myocardial ischemia, and other factors can produce a variety of cytokines (15), and most of them, especially the inflammatory cytokines, including tumor necrosis factor α (TNF-α), interleukin (IL)-6, IL-1β, and IL-10 are involved in the occurrence and development of VR (13, 16). The major inflammatory cytokines that promote VR are TNF-α, IL-1β, and IL-6. Tumor necrosis factor α and IL-1 may promote VR by its effects on cardiomyocytes, macrophages, and the extracellular matrix (ECM). In cardiomyocytes, TNF-α may trigger apoptosis by activating the inherent pathway of cell death (17, 18). In macrophages, TNF-α can stimulate the synthesis of other pro-inflammatory cytokines (17, 19). In white blood cells, IL-1 can activate white blood cells, and stimulate downstream inflammatory responses (17, 20, 21). In fibroblasts, TNF-α and IL-1 can disrupt the balance between matrix metalloproteinases (MMPs) and their inhibitors, leading to the degradation of the ECM (17). Moreover, TNF-α and IL-1 can induce the expression of endothelial adhesion molecules in the microvasculature, leading to the enhancement of adhesive interactions between circulating leukocytes and the endothelial cell lining. Because of this, the inflammatory cells may accumulate in the cardiac microcirculation, finally leading to tissue damage and cardiac dysfunction (17). Interleukin-6 plays different roles *via* the gp130/STAT3 pathways (17). Interleukin-6 can promote cardiac hypertrophy in cardiomyocytes (22). In fibroblasts, IL-6 promotes proliferation and stimulates ECM synthesis (23). Additionally, IL-6 can regulate the function of macrophages and lymphocytes (24). However, IL-10 can suppress the release of inflammatory mediators by monocyte macrophages, thus inhibiting the secretion of TNF-α, IL-1β, IL-1, IL-6, IL-8, granulocyte-colony stimulating factors, and granulocyte-macrophage-colony stimulating factors caused by lipopolysaccharide and interferon-γ (25–27). Furthermore, IL-10 enhances the anti-inflammatory factor release, such as IL-1 receptor antagonists and soluble TNF-α receptors (25–27).

Heart-Protecting Musk Pill, prepared using *Moschus* (Shexiang), *Panax ginseng C.A.Mey*. (Renshen), *Bos taurus domesticus Gmelin* (Niuhuang), *Cinnamomum cassia (L.) J.Presl* (Rougui), *Liquidambar orientalis Mill*. (Suhexiang), *Bufo bufo gargarizans Cantor* (Chansu), and *Borneol* (C_10_H_18_O, Bingpian), can inhibit inflammatory reactions and VR after acute myocardial infarction by reducing the levels of TNF-α and IL-6, leading to the increase of the maximum value of left ventricular systolic pressure and left ventricular end-systolic pressure and the reduction of the left ventricular end-diastolic pressure, consequently improving the left ventricular function in rats with acute myocardial infarction (28). Xin-Ji-Er-Kang Formula, composed of *Panax ginseng C.A.Mey*. (Renshen), *Polygonatum adnatum S.Yun Liang* (Yuzhu), *Panax pseudoginseng var. Notoginseng* (Burkill) *G. Hoo* & *C.L. Tseng* (Sanqi), *Allium macrostemon Bunge* (Xiebai), *Angelica sinensis* (Oliv.) *Diels* (Danggui), *Ophiopogon japonicus* (Thunb.) *Ker Gawl*. (Maidong), *Schisandra chinensis* (Turcz.) *Baill*. (Wuweizi), *Salvia miltiorrhiza Bunge* (Danshen), *Sophora flavescens Aiton* (Kushen), *Glycyrrhiza uralensis Fisch*. (Gancao), *Radix Astragali* (Huangqi), *Epimedium acuminatum Franch*. (Yinyanghuo), *Trichosanthes kirilowii Maxim*. (Gualou), and *Dryobalanopsaromatica C.F. Gaertn*. (Longnao), can reduce the expression of TNF-α and IL-1β, and increase the expression of IL-10 to restore the balance between the pro-inflammatory and anti-inflammatory state in mice with Angiotensin II (Ang II)-induced human umbilical vein endothelial cells injury (29). Yi-Qi-Wen-Yang Decoction, composed of *Radix Astragali* (Huangqi), *Sedum erythrostictum Miq*. (Jingtian), *Aconitum carmichaelii Debeaux* (Fuzi), *Polyporus umbellatus* (Pers.) *Fries* (Zhuling), *Cervus nippon Temminck* (Lurong), *Curcuma phaeocaulis Valeton* (Ezhu), *Paeonia lactiflora Pall*. (Baishao), and *Zingiber officinale Roscoe* (Shengjiang), was capable of inhibiting inflammatory responses through upregulating IL-10 and downregulating TNF-α (30).

The nuclear factor kappa B (NF-κB) transcriptional activation pathway is considered to be the main regulator of inflammation (31). Nuclear factor kappa B can bind to the inhibitor of kappa B (IκB) protein in the cytoplasm (32). Under the action of pro-inflammatory cytokines, IκB is phosphorylated and degraded, releasing NF-κB dimer, so that NF-κB can be transferred to the nucleus, which induces the transcription of many genes and leads to the expression of inflammatory proteins, such as TNF-α and IL-6 (33–35).

Qi-Li-Qiang-Xin, which consists of *Panax ginseng C.A.Mey*. (Renshen), *Radix Astragali* (Huangqi), *Aconitum carmichaelii Debeaux* (Fuzi), *Salvia miltiorrhiza Bunge* (Danshen), *Alisma plantago-aquatica subsp. orientale (Sam.) Sam*. (Zexie), *Carthamus tinctorius L*. (Honghua), *Polygonatum adnatum S.Yun Liang* (Yuzhu), *Citrus reticulata Blanco* (Chenpi), *Ramulus Cinnamomi* (Guizhi), and *Semen Lepidii/Semen Descurainiae* (Tinglizi), can reduce the inflammatory response in a rat model of myocardial infarction through inhibiting the activity of NF-κB, which is mainly by reducing the expression of NF-κB p65 in the nucleus and the phosphorylation of IκB (32). Moreover, Qi-Li-Qiang-Xin improved cardiac function, reduced left ventricular dimension, inhibited interstitial inflammation and fibrosis, increased neovascularization, and attenuated cardiomyocyte apoptosis through the upregulated hypoxia-inducible factor-1α (HIF-1α), vascular endothelial growth factor (VEGF), and enhanced phosphorylation of Akt (36). Qi-Shen-Yi-Qi Pill, which consists of *Radix Astragali* (Huangqi), *Salvia miltiorrhiza Bunge* (Danshen), *Panax pseudoginseng var. Notoginseng (Burkill) G. Hoo* & *C.L. Tseng* (Sanqi), and *Dalbergia odorifera T. Chen* (Jiangxiang), improved cardiac remodeling accompanied with a restoration of the Ang II-NADPH oxidase-reactive oxygen species (ROS)-MMPs pathways and reduction of the TNF-α/NFκB and IL-6/STAT3 pathways (37). Qing-Da Granule, composed of *Gastrodia elata Blume* (Tianma), *Scutellaria baicalensis Georgi* (Huangqin), *Uncaria rhynchophylla (Miq.) Miq. ex Havil*. (Gouteng), and *Nelumro nucifera Gaertn*. (Lianzixin), can reduce the infiltration of macrophages and the activation of proinflammatory cytokines (TNF-α, IL-6) by inhibiting the NF-κB pathway in spontaneously hypertensive rats (38).

### Oxidative Stress

Oxidative stress is due to the loss of balance of redox between the level of ROS and endogenous antioxidant capacity, which causes the relative deficiency of antioxidant capacity and the relative increase of intracellular ROS (39–41). The increase of some toxic ROS leads to the loss of cell function, gene mutation, and even death (14, 42). Studies have shown that ROS is related to myocardial infarction and myocardial hypertrophy (43, 44). The stimulation of VR by oxidative stress involves the activation of several downstream signal pathways. First, ROS can activate a variety of hypertrophic signal kinases and transcription factors, including mitogen-activated protein kinase (MAPK), c-Jun N-terminal kinase (JNK), p38, NF-κB, and Akt kinases, to promote cardiac hypertrophy and cardiomyocyte apoptosis (43, 45, 46). Second, ROS can activate poly [ADP-ribose] polymerase 1 (PARP-1) by causing DNA strand breaks. Poly [ADP-ribose] polymerase 1 can regulate the expression of many inflammatory mediators and promote the progress of cardiac remodeling (43). Third, ROS can cause calcium ion (Ca^2+^) overload, increasing the mitochondrial permeability, and leading to the loss of mitochondrial inner membrane transmembrane potential, and then results in cell death (43, 46). Fourth, ROS can stimulate the proliferation of cardiac fibroblasts, activate MMPs, and lead to ECM remodeling (46, 47).

Xanthine oxidase is the main source of ROS in the cardiovascular system, and the inhibition of its enzyme activity can greatly reduce the production of ROS (48, 49). Qi-Li-Qiang-Xin can significantly reduce the activity of serum xanthine oxidase in rats after myocardial infarction and enhance the ability of myocardial tissue to scavenge oxygen (O^2−^) and hydroxyl radicals (50). Under the condition of chronic intermittent hypoxia in mice, Sheng-Mai-San can increase the activities of antioxidant enzymes (superoxide dismutase and catalase) and reduce the contents of malondialdehyde and 4-HNE (51). In addition, Sheng-Mai-San can also reduce the level of serum myeloperoxidase (52).

As the main vascular endothelial relaxing factor, nitric oxide (NO) has the effect of relaxing vascular smooth muscles, scavenging free radicals, and inhibiting lipid peroxidation. However, TNF-α can decrease NO content, because TNF-α can not only activate the nicotinamide adenine dinucleotide phosphate (NADPH) oxidase and make the superoxide produced by it react with NO, but also can inhibit the initiator of endothelial NO synthases (eNOS) and disturb the stability of the eNOS gene, leading to the decrease of the activity level of eNOS and NO (53, 54). However, Xin-Ji-Er-Kang Formula could significantly decrease the level of TNF-α, inhibit the activation of NADPH oxidase, reduce the uncoupling of eNOS and the production of ROS, and increase the activity of eNOS and NO content, thus improving the function of vascular endothelium (29, 55). The Xin-Ji-Er-Kang Formula has blunted the decrease of superoxide dismutase, NO, and the increase in malondialdehyde and Ang II serum contents, myocardial cross-section area, collagen volume fraction, and perivascular circumferential collagen area compared with the hypertensive model group. It also reduced the serum content of hydroxyproline while increasing the tetrahydrobiopterin levels in cardiac tissue by suppressing the JNK/MAPK pathway (56). Yi-Qi-Fu-Mai Powder Injection, redeveloped from Sheng-Mai-San, can decrease ROS generation, malondialdehyde content, and increase NO contents to alleviate the injury of vascular endothelial cells induced by hypoxia/reoxygenation (57). The mitochondria are the main target and source of ROS. Excessive ROS will not only lead to the non-specific damage of cells but also lead to mitochondrial dysfunction (58, 59). Yi-Qi-Fu-Mai Powder Injection can reduce the production of ROS and improve mitochondrial function by down-regulating the expression of NADPH oxidase subunits, such as NOX2, p67 phox, and NOX4 (60). However, due to the lack of experimental data, it is not clear whether TCM can remove all types of ROS.

### Apoptosis Factors and Autophagy

Cardiomyocyte apoptosis is closely related to the occurrence and development of autophagy and VR (61). Caspase, Bcl-2, and Fas proteins are the major regulators of apoptosis. Many factors, such as AngII, inflammatory factors, and ROS, can promote the expression of apoptosis signals, and then promote apoptosis. Qi-Shen-Yi-Qi Pills could inhibit the expression of Fas ligand and p53 by activating the expression of murine double minute 2 and play an anti-apoptosis role in cardiomyocytes (62). Qi-Shen-Yi-Qi Pills could reduce inflammatory reaction by down-regulating the expression of inflammatory cytokines (TNF-α and IL-6) (62). Cyclooxygenase 2, which is highly linked with inflammatory cytokines, could be inhibited by Qi-Shen-Yi-Qi Pills to limit the conversion of arachidonic acid to prostaglandin E2 (PGE2), and we know that PGE2 and its receptors could induce apoptosis by increasing the transcriptional activity of the p53 gene and the expression of Fas ligand (63–65). Qi-Shen-Yi-Qi Pills could remarkably reduce the expression of PGE2 and its receptors (62). The proportion of Bax/Bcl-2 protein is the key factor to determine the inhibitory effect on apoptosis (66). The activation of the PI3K/Akt signal has been proved to prevent cardiomyocyte apoptosis and protect the myocardium (36). Through upregulating the expression of neuregulin-1, Qi-Li-Qiang-Xin can activate the PI3K/Akt signal pathway, promote Akt phosphorylation, stimulate the expression of vascular growth factor, and activate the anti-apoptosis protein, leading to the inhibition of the proportion of Bax/Bcl-2 and the expression of Caspase-3 (36). Yi-Qi-Wen-Yang Decoction suppressed the upregulated Bax, cleaved caspase-3 and PARP, and increased the downregulated Bcl-2 through activating the IL-10/Stat3 signaling and inactivating the NF-Kb P65 signaling to inhibit cardiomyocyte apoptosis (30). Qi-Li-Qiang-Xin can also inhibit cardiomyocyte apoptosis in non-infarcted zone rats by reducing the production of ROS (67). In addition, Qi-Li-Qiang-Xin can also reduce the expression of p53 (36, 67). By upregulating the expression of osteoprotegerin and tumor necrosis factor-related apoptosis-inducing ligand, Tian-Ma-Gou-Teng Decoction, made up of *Uncaria rhynchophylla (Miq.) Miq. ex Havil*. (Gouteng), *Gastrodia elata Blume* (Tianma), *Scutellaria baicalensis Georgi* (Huangqin), *Eucommia ulmoides Oliv*. (Duzhong), Achyranthes bidentata Blume (Niuxi), Cichlanthus chinensis (DC.) Tiegh. (Sangjisheng), *Haliotis diversicolor Reeve* (Shijueming), *Gardenia jasminoides J.Ellis* (Zhizi), *Leonurus japonicus Houtt*. (Yimucao), *Polygonum multiflorum Thunb*. (Heshouwu), and *Poria cocos (Schw.) Wolf* (Fuling), can activate p-Akt and inhibit caspase cascade to play the role of inhibiting cardiomyocyte apoptosis (68). Lu-Hong Formula, consisting of *Cervus nippon Temminck* (Lurong), *Carthamus tinctorius L*. (Honghua), *Radix Astragali* (Huangqi), *Codonopsis affinis Hook.f*. & *Thomson* (Dangshen), *Cinnamomum cassia (L.) J.Presl* (Rougui), and *Semen Lepidii/Semen Descurainiae* (Tinglizi) can reduce cardiomyocyte apoptosis by down-regulating the expression of caspase-3 (69). Huo-Xue-Qian-Yang Decoction, consisting of *Salvia miltiorrhiza Bunge* (Danshen), *Hirudo nipponica Whitman* (Shuizhi), *Uncaria rhynchophylla (Miq.) Miq. ex Havil*. (Gouteng), *Haliotis diversicolor Reeve* (Shijueming), *Crataegus pinnatifida Bunge* (Shanzha), and *Zea mays* L. (Yumixu), can reduce the endoplasmic reticulum stress and downregulate the activating transcription factor 6-C/EBP homologous protein signaling pathway to inhibit cardiac apoptosis to ameliorate VR (70).

Cell survival requires the participation of apoptosis and autophagy. The interaction between these two pathways is complex and critical. Autophagy can promote survival by inhibiting apoptosis under several different conditions, and sometimes lead to cell death by cooperating with apoptosis or as a supplement to apoptosis defects. Tong-Xin-Luo, Tong-Guan Capsules, and Qi-Dan-Li-Xin Pills can all play the role of anti-VR by anti-cardiomyocyte apoptosis and promoting cardiomyocyte autophagy (71–73). Rapamycin mechanical target is a key regulatory protein involved in protein production, cell growth/proliferation, autophagy, lysosomal function, and the metabolic pathway, and is considered to be the central regulator of cell growth. Tong-Xin-Luo phosphorylates TSC2 and RAPTOR by activating adenosine monophosphate (AMP)-activated protein kinase (AMPK), which leads to the decrease of rapamycin mechanical target (mTOR) phosphorylation and increasing cardiomyocyte autophagy (71). Tong-Guan Capsule promotes cardiomyocyte autophagy in the myocardial infarction mouse model by activating Sirt1 and down-regulating the mTOR/P70/S6K/4EBP1 pathway (72). It can also deacetylate p53 to inhibit apoptosis. Qi-Dan-Li-Xin Pill also promotes the autophagy of cardiomyocytes by down-regulating the mTOR/P70/S6K/4EBP1 pathway (73). Yang-Xin-Kang Tablets, prepared using *Panax ginseng C.A.Mey*. (Renshen), *Radix Astragali* (Huangqi), *Ophiopogon japonicus (Thunb.) Ker Gawl*. (Maidong), *Schisandra chinensis (Turcz.) Baill*. (Wuweizi), and *Ilex pubescens Hook*. & *Arn*. (Maodongqing), protects against myocardial injury after myocardial infarction by inhibiting the AMPK/mTOR signal pathway and excessive autophagy (74).

### TGF-β1

Transforming growth factor β may be the most characteristic fibrotic growth factor (75). Transforming growth factor β has three subtypes (TGF-β1, 2, and 3), encoded by three different genes. It is currently the subject of most studies, and its overexpression leads to myocardial fibrosis (13). In addition to TGF-β, the signaling pathways associated with TGF-β are also involved in myocardial fibrosis. The TGF-β/Smads signaling pathway is the classical pathway of myocardial fibrosis. The abnormal activation of the TGF-β/Smads signal pathway can promote the proliferation of fibroblasts and the production of collagen and ECM, leading to the process of the aggravation of the myocardial fibrosis (76, 77). Some research has shown that TGF-β and its signaling pathways can be activated by Ang II, ROS, and inflammatory factors (13, 17, 78, 79).

Lu-Hong Formula can inhibit the expression of TGF-β1, and then inhibit the expression of collagen type I and III, and fibronectin genes and proteins, leading to the inhibition of the proliferation and deposition of collagen in the left ventricle in pressure-overloaded rats (69). Guanxin V could inhibit the TGF-β1 pathway to exert an anti-VR effect (80). Bu-Yang-Huan-Wu Decoction, prepared using *Radix Astragali* (Huangqi), *Angelica sinensis (Oliv.) Diels* (Danggui), *Paeonia lactiflora Pall*. (Chishao), *Ligusticum chuanxiong* (Chuanxiong), *Prunus persica (L.) Batsch* (Taoren), *Carthamus tinctorius L*. (Honghua), and *Lumbricus* (Dilong), can inhibit the activation of Smad3 and Smad4, which then inhibit the transcription of pro-fibrotic molecules, including the transcription of α-smooth muscle actin, collagen, and tissue inhibitors of MMPs, and finally, reduce the activation of myofibroblasts and matrix deposition, leading to the alleviation of pressure overload-induced cardiac remodeling (81). Dan-Qi soft capsules, Tong-Guan capsules, and Qi-Li-Qiang-Xin can inhibit the differentiation and formation of myofibroblasts by inhibiting the TGF-β1/Smad3 pathway, having an effect of inhibiting VR (32, 82, 83). Moreover, Qi-Li-Qiang-Xin may also promote the TGF-β3/Smad7 signal pathway to play an anti-VR effect (84). Ling-Gui-Zhu-Gan Decoction, composed of *Poria cocos (Schw.) Wolf* (Fuling), *Ramulus Cinnamomi* (Guizhi), *Atractylis macrocephala (Koidz.) Hand.-Mazz*. (Baizhu), and *Glycyrrhiza uralensis Fisch*. (Gancao), can significantly improve the pathological changes of the myocardial tissues, increase the left ventricular systolic pressure, left ventricular pressure maximum contraction rate, and left ventricular pressure maximum relaxation rate (85). Besides this, it can also decrease the left ventricular end-diastolic pressure and reduce the whole heart weight index and left ventricular weight index in rats with acute myocardial infarction (85). All of these are achieved by regulating the TGF-β/Smads pathway (85). Xin-Fu-Li Granule is a compound TCM that consists of extracts from *Radix Astragali* (Huangqi), *Panax ginseng C.A.Mey*. (Renshen), *Salvia miltiorrhiza Bunge* (Danshen), *Ligusticum chuanxiong* (Chuanxiong), *Alisma plantago-aquatica subsp. orientale (Sam.) Sam*. (Zexie), *Angelica sinensis (Oliv.) Diels* (Danggui), *Semen Lepidii/Semen Descurainiae* (Tinglizi), *Chaenomeles speciosa (Sweet) Nakai* (Mugua), *Areca catechu L*. (Binglang), and *Ophiopogon japonicus (Thunb.) Ker Gawl*. (Maidong), and can improve ventricular reconstruction and inhibit myocardial fibrosis in rats with acute myocardial infarction by regulating the TGF-β/Smads pathway (86).

What we should pay attention to is that TGF-β can also activate several other non-classical pathways, such as ras/ methyl ethyl ketone (MEK)/ extracellular signal-regulated kinases (ERK), p38, and JNK (78). The Si-Miao-Yong-An Decoction can significantly improve cardiac function and inhibit myocardial fibrosis in pressure-overloaded rats by inhibiting TGF-β1/Smad and TGF-β1/Tak1/p38 signal pathway (87). The connective tissue growth factor (CTGF) is the downstream effect factor of TGF-β1 and only mediates the negative effect of TGF-β1 (88, 89). Transforming growth factor β1 can induce the expression of CTGF, and CTGF can also enhance the TGF-β1 signal pathway (90, 91). Finally, a vicious circle is formed, resulting in the accumulation of ECM, and myocardial fibrosis occurs. Qi-Shen-Yi-Qi pills can inhibit the expression of CTGF protein and mRNA by regulating the TGF-β1/CTGF pathway and reducing the myocardial collagen deposition, leading to the improvement of VR on experimental autoimmune myocarditis rats (92).

Matrix metalloproteinases, acting as an activator in TGF-β (13), also play an important role in myocardial fibrosis. The abnormal increase and excessive deposition of a myocardial ECM plays a very important role in the occurrence and development of myocardial hypertrophy and myocardial fibrosis. Matrix metalloproteinases are a family of zinc-dependent proteases, which mainly participate in the metabolism of ECM and can reduce and release almost all ECM components except polysaccharides (93). After tissue damage, the regulation of MMPs activity is out of control, the ratio of MMPs to tissue inhibitor of metalloproteinase is out of balance, and myocardial ECM accumulates excessively, which leads to myocardial fibrosis and VR (94–96). Si-Miao-Yong-An Decoction can increase the expression of MMP9 and decrease the expression of TIMP2 to promote the degradation of collagen and reduce the synthesis of collagen, leading to the inhibition of myocardial fibrosis (87). Dan-Shen Injection can prevent left VR by inhibiting the activation of MMP2 and MMP9 to improve ejection fraction and left ventricular stroke volume in rats with myocardial infarction (97).

### Nuroendocrine System

#### SNS

Sympathetic activity is one of the main exogenous factors in the regulation of VR. After any type of myocardial injury, adrenaline can be activated instantly, which is the main means of increasing the heart rate and contractility and playing the role of stabilizing cardiac function (98). However, long-term activation will lead to VR. The increase of catecholamine secretion can cause cardiomyocyte necrosis and apoptosis due to hypoxia, increased sarcolemmal permeability, calcium overload, the elevation of cyclic AMP (cAMP), and formation of oxidative catecholamine metabolites (98–101). Norepinephrine can stimulate fetal gene reprogramming, promote fibroblasts and protein synthesis, and aggravate VR (100, 102). Sheng-Mai-San, consisted of *Panax ginseng C.A.Mey*. (Renshen), *Ophiopogon japonicus (Thunb.) Ker Gawl*. (Maidong), and *Schisandra chinensis (Turcz.) Baill*. (Wuweizi), can reduce the contents of norepinephrine and 5-hydroxytryptamine in rats after myocardial infarction by regulating the activity of the SNS, and then the myocardial contraction and heart rate were slowed down (103). Additionally, Sheng-Mai-San may inhibit the activation of the hypothalamic-pituitary-adrenal axis by reducing the levels of IL-6 and TNF-α in patients with heart failure, and then control the activity of SNS and the secretion of neuropeptides. Qing-Da Granule, composed of *Gastrodia elata Blume* (Tianma), *Scutellaria baicalensis Georgi* (Huangqin), *Uncaria rhynchophylla (Miq.) Miq. ex Havil*. (Gouteng), and *Nelumro nucifera Gaertn*. (Lianzixin), can dilate blood vessels, reduce vascular tension, and inhibit vascular remodeling by suppressing the activation of the L-type Ca^2+^channel and inhibiting the influx of Ca^2+^ (104). Related research has shown that Ca^2+^ plays an important role in the SNS (105). The generation of sympathetic activity depends on the action potential produced by the influx of Ca^2+^, so the inhibition of Ca^2+^ influx can inhibit the sympathetic nerve activity (101, 106).

#### RAAS

The RAAS plays a very important role in the occurrence and development of VR (107, 108). The chronic activation of RAAS leads to a long-term increase in the levels of Ang II and aldosterone, both of which are involved in pathological processes including cardiomyocyte hypertrophy, interstitial fibrosis, and cardiomyocyte apoptosis (109, 110). Ang II, the main effector of RAAS, can not only activate the SNS and increase the heart rate and contractility but also cause systemic vasoconstriction which leads to an increase in the total peripheral resistance and aggravate cardiac load (110, 111). Ang II can stimulate different cytokines, such as endothelin-1 (ET-1), aldosterone, brain natriuretic peptide (BNP), TNF-α, and IL, and these cytokines are involved in the process of VR *via* different ways (112–116). Ang II can induce ET-1 by ROS and ERK (78, 117), and ET-1 can not only contract blood vessels but also induce fibroblasts to produce ECM (118). Ang II can induce aldosterone production in the adrenal cortex, and aldosterone can induce cardiomyocyte apoptosis and cardiomyocyte hypertrophy by activating oxidative stress and regulating ion channels (119, 120). Related research has shown that Ang II can increase the secretion of TNF-α and IL-6 (121), and the mechanism of their participation in VR was described in the following paragraphs. Ang II can stimulate the release of natriuretic peptides (122, 123). The long-term overstimulation of RAAS in chronic heart failure promotes increased sodium and water retention, which leads to increased pressure in the left ventricle and atrium, and finally stimulates the synthesis and secretion of BNP (123). Moreover, Ang II can upregulate the TGF-β1 expression by binding to Ang II type 1 receptors (AT1R) (78, 124), and then the TGF-β/Smads signal pathway is activated which is related to myocardial fibrosis (78).

Guanxin V consists of *Codonopsis affinis Hook.f*. & *Thomson* (Dangshen), *Ophiopogon japonicus (Thunb.) Ker Gawl*. (Maidong), *Schisandra chinensis (Turcz.) Baill*. (Wuweizi), *Rehmannia chingii H.L. Li* (Dihuang), *Salvia miltiorrhiza Bunge* (Danshen), and *Paeonia lactiflora Pall*. (Chishao) (80, 125) and has a significant effect on VR (126). Mechanistically, in an animal model, Guanxin V can inactivate RAAS in VR after acute myocardial infarction through the non-angiotensin converting enzyme pathway, which significantly reduces the level of Ang II, reduces the infarct size after acute myocardial infarction, protect cardiac function, reduce myocardial fibrosis, and thus, reduce VR (127). Poge Heart-Saving Decoction, prepared with *Aconitum carmichaelii Debeaux* (Fuzi), *Zingiber officinale Roscoe* (Ganjiang), *Glycyrrhiza uralensis Fisch*. (Gancao), *Cornus officinalis Siebold* & *Zucc*. (Shanzhuyu), *Os Draconis* (Longgu), *Concha Ostreae* (Muli), *Magnetitum* (Cishi), *Panax ginseng C.A.Mey*. (Renshen), and *Moschus* (Shexiang), can reduce the left ventricular end-diastolic dimension and left ventricular end-systolic dimension by inhibiting the level of RAAS, especially aldosterone and Ang II levels, and finally, decrease the post-load of the heart and increase the ejection fraction, reverse VR, and improve cardiac function (128). Jiajian Yu-Nv-Jian, containing *Gypsum* (CaSO_4_·2H_2_O, Shigao), *Anemarrhena asphodeloides Bunge* (Zhimu), *Scrophularia microdonta Franch*. (Xuanshen), *Rehmannia chingii H.L. Li* (Dihuang), and *Ophiopogon japonicus (Thunb.) Ker Gawl*. (Maidong), can significantly reduce the experimental cardiac remodeling by improving hemodynamics and inhibiting the activation of RAAS. It can also reduce the production of ET-1 and the contents of Ang II, aldosterone, and hydroxyproline, and down-regulate the expression of AT1R, TNF-α, and TGF-β1 (113). Morphologically, Jiajian Yu-Nv-Jian decreased the cross-sectional area of cardiomyocytes, collagen volume fraction, collagen types I and III, and the perivascular collagen area (113). RAAS mediates the development of VR mainly by inducing the generation of Ang II, and TCM has confirmed that it can inhibit the level of Ang II not only by classical RAAS but also by the non-angiotensin converting enzyme pathway. However, we do not figure out the mechanism of Ang II produced by the non-angiotensin converting enzyme pathway.

### PPARγ

PPARγ is a nuclear receptor that can change the transcription of many target genes (129, 130). It is mainly related to energy metabolism. Peroxisome proliferator-activated receptor γ can stimulate the transcription of genes related to lipid metabolism and promotes adipocyte differentiation. Moreover, PPARγ can also suppress the production of proinflammatory cytokines and then inhibit proliferation and migration (130). The PPARγ coactivator 1α (PGC-1α) is the main regulator of lipid catabolism, oxidative metabolism, mitochondrial metabolism, and biogenesis-related genes, reflecting the dysfunction of the mitochondria, which plays an important role in the control of myocardial metabolism (131–133). High levels of PGC-1α may be related to higher levels of oxidative metabolism, higher oxygen consumption, and lower general oxidative stress, while lower levels of PGC-1α may be related to increased dependence on glycolysis, lower oxygen consumption, and higher ROS levels (131, 132). By upregulating the expression of PPARα and PPARγ, Shen-Qi-Fu-Zheng Injection extracted from *Codonopsis affinis Hook.f*. & *Thomson* (Dangshen) and *Radix Astragali* (Huangqi), can interfere with the metabolic process of the injury, inhibit ischemic cardiac structural and functional disorders such as myocardial hypertrophy and VR, effectively improve the damaged cardiac function, and achieve the protective effect of ischemia/reperfusion injury (134). Qi-Li-Qiang-Xin can improve cardiac energy metabolism by up-regulating PGC-1α and PPARγ, which alleviate myocardial hypertrophy and cardiac remodeling, and significantly improve cardiac function, including ejection fraction and fraction shortening (135–138). However, due to the lack of sufficient experimental data, we do not yet understand the mechanism of TCM suppressing PPARγ and PGC-1α.

## Discussion

Ventricular remodeling is a process of a series of morphological and structural changes in cardiomyocytes, collagen grids, and vascular beds, which is the basic mechanism of heart failure, so it is of great significance to study the pathogenesis of VR for the prevention and treatment of heart failure.

The inflammatory response is one of the key factors in VR. To promote the repair of injured areas, the damaged myocardium will release its intracellular contents and cause inflammation by activating the innate immune mechanism. Neutrophils were first recruited, followed by pro-inflammatory monocytes/macrophages and lymphocytes (11, 12). These cells remove dead cells and matrix fragments from the damaged areas through different pathways and then activate the repair pathways needed for scar formation. However, the aggravation, prolongation, or expansion of the inflammatory response can lead to more severe remodeling and dysfunction. Excessive early inflammation may increase matrix degradation, leading to heart rupture (11). Prolonged inflammation may damage collagen deposition, resulting in the formation of scars with reduced tensile strength, and finally, the increase of the chamber dilatation (11). The enhanced expression of pro-inflammatory mediators can stimulate the production of ROS, activating the pro-apoptosis pathway, and inducing further loss of cardiomyocytes (43). Finally, defective control of inflammatory response may lead to inflammatory infiltration extending into the non-infarcted myocardium, enhancing fibrosis and worsening the diastolic function (11, 17). In addition, due to the overload of antioxidant defense in the damaged heart, ROS production, and induced inflammatory signals, such as IL-1 β, directly inhibit myocardial function (46).

To maintain the basic function of the damaged heart, SNS and RAAS can be activated. However, the excessive activation of SNS will lead to the excessive accumulation of catecholamines, which is linked to cardiomyocyte apoptosis and myocardial fibrosis. The activation of RAAS will cause too much Ang II generation and it can participate in a variety of reactions. Ang II can promote the release of inflammatory cytokines and ROS through AT1R (139). In addition, Ang II is involved in myocardial fibrosis. Ang II can up-regulate the expression of TGF-β, which plays an important role in VR (139). Transforming growth factor -β can not only increase the production of ECM but also upregulate the expression of mesenchymal makers by activating the TGF-β/Smad3 signal pathway, leading to the formation of myofibroblasts (78, 139). What is more, Ang II can also promote the production of aldosterone, which can upregulate the expression of α-smooth muscle actin and promote myocardial fibrosis (139).

In addition to the mechanism described above, the energy metabolism of cardiomyocytes also affects the process of VR. Abnormal energy metabolism will lead to ROS production and lead to a series of chain reactions, such as inflammation, activation of the neuroendocrine system, and cardiomyocyte apoptosis, thus, improving the myocardial energy metabolism is helpful to improve VR.

Because of its multi-components and multi-targets, TCM has good effects on the prevention and treatment of VR. From the literature we enumerated, Qi-Li-Qiang-Xin is the most studied drug. Based on a variety of studies on Qi-Li-Qiang-Xin, we can find that it can exert the effect of anti-myocardial apoptosis and myocardial fibrosis by inhibiting the production of inflammatory factors, reducing the production of ROS, up-regulating PGC-1α and PPARγ, inhibiting the TGF-β1/Smad3 pathway, and activating the TGF-β3/Smad7 signal pathway. Other TCM, like Qi-Shen-Yi-Qi, can also reduce VR by reducing inflammation and inhibiting the TGF-β1/CTGF pathway. Traditional Chinese medicine improves VR mainly by acting on cardiomyocytes, fibroblasts, and inflammatory cells, but due to the limitations of related experiments, it is not clear what kind of inflammatory cells TCM can reduce. From Table 1, we can find that many TCM has only done related research on one of the mechanism mentioned above, which is not beneficial to our further understanding of the anti-VR mechanism of TCM. Perhaps we can speculate the multiple mechanisms of anti-VR from the same components of different TCM, but we do not know whether the previous interactions of different TCM have changed the structure of the active components of TCM, which requires us to further improve the basic research of TCM against VR.

**Table 1:** Details of constituents and mechanism of traditional Chinese medicine (TCM) for ventricular remodeling (VR).

**TCM**	**Constituents**	**Mechanism**	**Cell type**	**References**
Guanxin V	*Codonopsis affinis Hook.f*. & *Thomson* (Dangshen), *Ophiopogon japonicus (Thunb.) Ker Gawl*. (Maidong), *Schisandra chinensis (Turcz.) Baill*. (Wuweizi), *Rehmannia chingii H.L. Li* (Dihuang), *Salvia miltiorrhiza Bunge* (Danshen), and *Paeonia lactiflora Pall*. (Chishao)	Inactivate RAAS through non-angiotensin converting enzyme pathway, which significantly reduce the level of Ang II, reduce the infarct size, protect cardiac function, reduce myocardial fibrosis, and inhibit TGF-β1 pathway	Myocardial cell and fibroblast	(127)
Poge Heart-Saving Decoction	*Aconitum carmichaelii Debeaux* (Fuzi), *Zingiber officinale Roscoe* (Ganjiang), *Glycyrrhiza uralensis Fisch*. (Gancao), *Cornus officinalis Siebold* & *Zucc*. (Shanzhuyu), *Os Draconis* (Longgu), *Concha Ostreae* (Muli), *Magnetitum* (Cishi), *Panax ginseng C.A.Mey*. (Renshen), and *Moschus* (Shexiang)	Reduce left ventricular end-diastolic dimension and left ventricular end-systolic dimension by inhibiting the level of RAAS, and finally decrease the postload of the heartand, increase ejection fraction, reverse VR, and improve cardiac function	Myocardial cell and fibroblast	(128)
Jiajian Yu-Nv-Jian	*Gypsum* (CaSO_4_·2H_2_O, Shigao), *Anemarrhena asphodeloides Bunge* (Zhimu), *Scrophularia microdonta Franch*. (Xuanshen), *Rehmannia chingii H.L. Li* (Dihuang), and *Ophiopogon japonicus (Thunb.) Ker Gawl*. (Maidong)	Improve hemodynamics, inhibit the activation of RAAS, down-regulate the expression of AT1R, TNF-α, and TGF-β1, and decrease cross-sectional area of cardiomyocyte, collagen volume fraction, collagen types I and III, and perivascular collagen area	Myocardial cell and fibroblast	(113)
Qi-Li-Qiang-Xin	*Panax ginseng C.A.Mey*. (Renshen), *Radix Astragali* (Huangqi), *Aconitum carmichaelii Debeaux* (Fuzi), *Salvia miltiorrhiza Bunge* (Danshen), *Alisma plantago-aquatica subsp. orientale (Sam.) Sam*. (Zexie), *Carthamus tinctorius L*. (Honghua), *Polygonatum adnatum S.Yun Liang* (Yuzhu), *Citrus reticulata Blanco* (Chenpi), *Ramulus Cinnamomi* (Guizhi), and *Semen Lepidii/Semen Descurainiae* (Tinglizi)	Reduce the inflammatory response though NF-κB pathway; improve cardiac function, reduce left ventricular dimension, inhibit interstitial inflammation and fibrosis, increase neovascularization, and attenuate apoptosis though upregulated HIF-1α, VEGF and enhanced p-Akt; activate PI3K/Akt signal pathway, stimulate the expression of vascular growth factor, and activate anti-apoptosis protein and inhibit the proportion of Bax/Bcl-2 and the expression of Caspase3 by up-regulating the expression of neuregulin-1; inhibit cardiomyocyte apoptosis by reducing the production of ROS and the expression of p53; reduce the activity of xanthine oxidase and enhance the ability to scavenge O^2−^ and hydroxyl radical; improve cardiac energy metabolism by up-regulating PGC-1α and PPARγ, and significantly improve cardiac function, including ejection fraction and fraction shortening; inhibit TGF-β1/Smad3 pathway; and activate TGF-β3/Smad7 signal pathway	Myocardial cell, endothelial cell, fibroblast, and inflammatory cell	(32, 36, 50, 67, 84, 135–138)
Qi-Shen-Yi-Qi Pill	*Radix Astragali* (Huangqi), *Salvia miltiorrhiza Bunge* (Danshen), *Panax pseudoginseng var. Notoginseng (Burkill) G. Hoo* & *C.L. Tseng* (Sanqi), and *Dalbergia odorifera T. Chen* (Jiangxiang)	Recover Ang II-NADPH oxidase-ROS-MMPs pathways and reduction of TNF-α/NFκB and IL-6/STAT3 pathways; inhibit the expression of CTGF, and reduce myocardial collagen deposition *via* TGF-β1/CTGF pathway; inhibit the expression of FasL and p53 by activating the expression of MDM2 to play an anti-apoptosis role; reduce inflammatory reaction by down-regulating the expression of TNF-α and IL-6; inhibit Cyclooxygenase 2 to limit the conversion of arachidonic acid to PGE2; reduce the expression of Prostaglandin E2 and its receptors	Myocardial cell and inflammatory cell	(37, 62, 92)
Qing-Da Granule	*Gastrodia elata Blume* (Tianma), *Scutellaria baicalensis Georgi* (Huangqin), *Uncaria rhynchophylla (Miq.) Miq. ex Havil*. (Gouteng), and *Nelumro nucifera Gaertn*. (Lianzixin)	Suppress the activity of the SNS by inhibiting the influx of Ca^2+^; reduce the infiltration of macrophages and inactivte TNF-α, IL-6 by inhibiting NF-κB pathway	Myocardial cell and macrophage	(38)
Heart-Protecting Musk Pill	*Moschus* (Shexiang), *Panax ginseng C.A.Mey*. (Renshen), *Bos taurus domesticus Gmelin* (Niuhuang), *Cinnamomum cassia (L.) J.Presl* (Rougui), *Liquidambar orientalis Mill*. (Suhexiang), *Bufo bufo gargarizans Cantor* (Chansu), and *Borneol* (C_10_H_18_O, Bingpian)	Inhibit the inflammatory reaction by reducing TNF-α and IL-6, increase the maximum value of left ventricular systolic pressure and left ventricular end-systolic pressure, and reduce left ventricular end-diastolic pressure, and improve left ventricular function	Myocardial. cell and inflammatory cell	(28)
Xin-Ji-Er-Kang Formula	*Panax ginseng C.A.Mey*. (Renshen), *Polygonatum adnatum S.Yun Liang* (Yuzhu), *Panax pseudoginseng var. Notoginseng (Burkill) G. Hoo* & *C.L. Tseng* (Sanqi), *Allium macrostemon Bunge* (Xiebai), *Angelica sinensis (Oliv.) Diels* (Danggui), *Ophiopogon japonicus (Thunb.) Ker Gawl*. (Maidong), *Schisandra chinensis (Turcz.) Baill*. (Wuweizi), *Salvia miltiorrhiza Bunge* (Danshen), *Sophora flavescens Aiton* (Kushen), *Glycyrrhiza uralensis Fisch*. (Gancao), *Radix Astragali* (Huangqi), *Epimedium acuminatum Franch*. (Yinyanghuo), *Trichosanthes kirilowii Maxim*. (Gualou), and *Dryobalanopsaromatica C.F. Gaertn*. (Longnao)	Reduce TNF-α and IL-1β, and increase IL-10 to restore the balance between the pro-inflammatory and anti-inflammatory state; decrease TNF-α, inhibit the activation of NADPH oxidase, reduce the uncoupling of eNOS and the production of ROS, and increase the activity of eNOS and NO content; blunt the decrease of superoxide dismutase, NO and the increase in malondialdehyde and Ang II, myocardial cross-section area, collagen volume fraction and perivascular circumferential collagen area; reduce hydroxyproline while increase tetrahydrobiopterin though suppressing JNK/MAPK pathway	Myocardial cell and endothelial cell	(29, 55, 56)
Yi-Qi-Wen-Yang Decoction	*Radix Astragali* (Huangqi), *Sedum erythrostictum Miq*. (Jingtian), *Aconitum carmichaelii Debeaux* (Fuzi), *Polyporus umbellatus (Pers.) Fries* (Zhuling), *Cervus nippon Temminck* (Lurong), *Curcuma phaeocaulis Valeton* (ezhu), *Paeonia lactiflora Pall*. (Baishao), and *Zingiber officinale Roscoe* (Shengjiang)	Attenuate myocardial inflammation, fibrosis, apoptosis, and reverse the impairment of cardiac function by activating the IL-10/Stat3 signaling pathway	Myocardial cell, fibroblast, and inflammatory cell	(30)
Bu-Yang-Huan-Wu Decoction	*Radix Astragali* (Huangqi), *Angelica sinensis (Oliv.) Diels* (Danggui), *Paeonia lactiflora Pall*. (Chishao), *Ligusticum chuanxiong* (Chuanxiong), *Prunus persica (L.) Batsch* (Taoren), *Carthamus tinctorius L*. (Honghua), and *Lumbricus* (Dilong)	Inhibit the activation of Smad3 and Smad4, then the pro-fibrotic molecules, including transcription of α-smooth muscle actin, collagen and tissue inhibitor of MMPs	Myocardial cell and inflammatory cell	(81)
Dan-Qi Soft Capsule	*Salvia miltiorrhiza Bunge* (Danshen), and *Panax pseudoginseng var. Notoginseng (Burkill) G. Hoo* & *C.L. Tseng* (Sanqi)	Inhibit TGF-β1/Smad3 pathway	Myocardial cell and fibroblast.	(82)
Tong-Guan Capsule	*Radix Astragali* (Huangqi), *Salvia miltiorrhiza Bunge* (Danshen), *Hirudo medicinalis* (Shuizhi) and *Ophiopogon japonicus (Thunb.) Ker Gawl*. (Maidong)	Inhibit the differentiation and formation of myofibroblasts; inhibit apoptosis and promote autophagy by activating Sirt1 and down-regulating mTOR/P70/S6K/4EBP1 pathway; deacetylate p53 to inhibit apoptosis	Myocardial cell and inflammatory cell	(72, 83)
Ling-Gui-Zhu-Gan Decoction	*Poria cocos (Schw.) Wolf* (Fuling), *Ramulus Cinnamomi* (Guizhi), *Atractylis macrocephala (Koidz.) Hand.-Mazz*. (Baizhu), and *Glycyrrhiza uralensis Fisch*. (Gancao)	Regulate TGF-β/Smads pathway	Myocardial cells and fibroblast	(85)
Xin-Fu-Li Granule	*Radix Astragali* (Huangqi), *Panax ginseng C.A.Mey*. (Renshen), *Salvia miltiorrhiza Bunge* (Danshen), *Ligusticum chuanxiong* (Chuanxiong), *Alisma plantago-aquatica subsp. orientale (Sam.) Sam*. (Zexie), *Angelica sinensis (Oliv.) Diels* (Danggui), *Semen Lepidii/Semen Descurainiae* (Tinglizi), *Chaenomeles speciosa (Sweet) Nakai* (Mugua), *Areca catechu L*. (Binglang), and *Ophiopogon japonicus (Thunb.) Ker Gawl*. (Maidong)	Regulate TGF-β/Smads pathway	Myocardial cell and fibroblast	(86)
Lu-Hong Formula	*Cervus nippon Temminck* (Lurong), *Carthamus tinctorius L*. (Honghua), *Radix Astragali* (Huangqi), *Codonopsis affinis Hook.f*. & *Thomson* (Dangshen), *Cinnamomum cassia (L.) J.Presl* (Rougui), and *Semen Lepidii/Semen Descurainiae* (Tinglizi)	Regulate TGF-β/Smads pathway and reduce apoptosis by down-regulating caspase-3	Myocardial cell and fibroblast	(69, 69)
Si-Miao-Yong-An Decoction	*Lonicera japonica Thunb*. (Jinyinhua), *Scrophularia microdonta Franch*. (Xuanshen), *Angelica sinensis (Oliv.) Diels* (Danggui), and *Glycyrrhiza uralensis Fisch*. (Gancao)	Inhibit TGF-β1/Smad and TGF-β1/Tak1/p38 signal pathways, and increase MMP9 and decrease TIMP2 to promote the degradation of collagen and reduce the synthesis of collagen	Fibroblast	(87)
Dan-Shen Injection	*Salvia miltiorrhiza Bunge* (Danshen)	Inhibit the activation of MMP2 and MMP9 to improve ejection fraction and left ventricular stroke volume	Myocardial cell, neutrophil, and macrophage	(97)
Sheng-Mai-San	*Panax ginseng C.A.Mey*. (Renshen), *Ophiopogon japonicus (Thunb.) Ker Gawl*. (Maidong), and *Schisandra chinensis (Turcz.) Baill*. (Wuweizi)	Inhibit the activity of the SNS and reduce the release of NE and 5-HT, increase the activity of antioxidant enzymes, and reduce malondialdehyde, 4-HNE, and myeloperoxidase	Myocardial cell	(51, 52)
Yi-Qi-Fu-Mai Powder Injection	*Panax ginseng C.A.Mey*. (Renshen), *Ophiopogon japonicus (Thunb.) Ker Gawl*. (Maidong), and *Schisandra chinensis (Turcz.) Baill*. (Wuweizi)	Decrease ROS generation, malondialdehyde content, and increase NO content; improve mitochondrial function by down-regulating the expression of NADPH oxidase subunits	Myocardial cell and endothelial cell	(57, 60)
Tong-Xin-Luo	*Panax ginseng C.A.Mey*. (Renshen), Buthus martensii Karsch (Quanxie), *Hirudo nipponica Whitman* (Shuizhi), *Eupolyphaga seusteleophage* (Tubiechong), *Scolopendra subspinipes* (Wugong), *Periostracum cicadae* (Chantui), *Paeonia lactiflora Pall*. (Chishao), Ziziphus jujuba Mill. (Suanzaoren), *Dalbergia odorifera T. Chen* (Jiangxiang), Santalum album L. (Tanxiang), and *Borneol* (C_10_H_18_O, Bingpian)	Inhibit apoptosis and promot autophagy though AMPK/mTOR pathway	Myocardial cell	(71)
Qi-Dan-Li-Xin Pill	*Radix Astragali* (Huangqi), *Salvia miltiorrhiza Bunge* (Danshen), *Epimedium acuminatum Franch*. (Yinyanghuo), and *Poria cocos (Schw.) Wolf* (Fuling)	Inhibit apoptosis and promote autophagy by down-regulating mTOR/P70/S6K/4EBP1 pathway	Myocardial cell	(73)
Yang-Xin-Kang Tablet	*Panax ginseng C.A.Mey*. (Renshen), *Radix Astragali* (Huangqi), *Ophiopogon japonicus (Thunb.) Ker Gawl*. (Maidong), *Schisandra chinensis (Turcz.) Baill*. (Wuweizi), and *Ilex pubescens Hook*. & *Arn*. (Maodongqing)	Inhibit AMPK/mTOR signal pathway and excessive autophagy	Myocardial cell	(74)
Tian-Ma-Gou-Teng Decoction	*Uncaria rhynchophylla (Miq.) Miq. ex Havil*. (Gouteng), *Gastrodia elata Blume* (Tianma), *Scutellaria baicalensis Georgi* (Huangqin), *Eucommia ulmoides Oliv*. (Duzhong), Achyranthes bidentata Blume (Niuxi), Cichlanthus chinensis (DC.) Tiegh. (Sangjisheng), *Haliotis diversicolor Reeve* (Shijueming), *Gardenia jasminoides J.Ellis* (Zhizi), *Leonurus japonicus Houtt*. (Yimucao), *Polygonum multiflorum Thunb*. (Heshouwu), and *Poria cocos (Schw.) Wolf* (Fuling)	Up-regulate osteoprotegerin and TNF-related apoptosis-inducing ligand, activate p-Akt and inhibit caspase cascade	Endothelial cell	(68)
Huo-Xue-Qian-Yang Decoction	*Salvia miltiorrhiza Bunge* (Danshen), *Hirudo nipponica Whitman* (Shuizhi), *Uncaria rhynchophylla (Miq.) Miq. ex Havil*. (Gouteng), *Haliotis diversicolor Reeve* (Shijueming), *Crataegus pinnatifida Bunge* (Shanzha), and *Zea mays L*. (Yumixu)	Reduce endoplasmic reticulum stress and down-regulate activating transcription factor 6-C/EBP homologous protein signaling pathway to inhibit cardiac apoptosis	Myocardial cell	(70)
Shen-Qi-Fu-Zheng Injection	*Codonopsis affinis Hook.f*. & *Thomson* (Dangshen) and *Radix Astragali* (Huangqi)	Interfere with the metabolic process of injury, inhibit ischemic cardiac structural and functional disorders, and effectively improve the damaged cardiac function	Myocardial cell	(134)

## Conclusion

The occurrence of VR is the result of the joint action of a variety of mechanisms (Table 2). In recent years, with the popularity of TCM, the advantages of TCM in the treatment of VR are more and more obvious (140). We do not comment on any monomer here, because the TCM formula consists of dozens of ingredients with numerous chemical molecules, making it difficult to elucidate the therapeutic mechanism of TCM (141–144). Through our research, we verify that TCM inhibits the process of VR through the single or multiple pathways and combined use of multiple drugs again. Some TCM, such as Qi-Li-Qiang-Xin, Qi-Shen-Yi-Qi Pill, Xin-Ji-Er-Kang Formula, and Yi-Qi-Wen-Yang Decoction, could play a role in many pathological conditions, including apoptosis, oxidative stress, and inflammation. From the above discussion, because of its multi-components and multi-action targets, TCM can inhibit VR effectively. However, the conclusion we got is based on a summary of numerous basic experiments, and this just provides evidence for subsequent clinical applications. We still need extensive clinical trials to assess the effectiveness and safety of TCM. Besides this, due to the holistic view and syndrome differentiation of TCM, we need to use different TCM formulas for patients with different syndromes, and we cannot establish treatment standards, which greatly increases the difficulty of clinical trials. In addition, the diversity of components also shows that it is difficult to identify the precise targets, which requires us to continue to improve the relevant technology.

**Table 2:** Details of the different mechanism on the heart.

**Item**	**Mechanism**
Inflammatory signals	Change the size, alter the shape, and affect the function of the heart
Oxidative stress	Affect the function of the heart
Apoptosis factors and autophagy	Affect the function of the heart
TGF-β1	Change the size, alter the shape, and affect the function of the heart
Nuroendocrine system	Change the size, alter the shape, and affect the function of the heart
PPARγ	Affect the function of the heart

## Author Contributions

Y-CZ, BL, and NG designed the study, acquired and researched the data for the article, and discussed its content. Y-CZ wrote the manuscript. NG and BL revised the manuscript. All authors read and approved the final manuscript.

## Funding

This work was partly supported by the National Natural Science Foundation of China (81774229), Research and Practice Innovation Plan for Postgraduates of Jiangsu, China (KYCX21_1641), Jiangsu Universities Nursing Advantage Discipline Project (2019YSHL095), and Jiangsu Leading Talent Project of Traditional Chinese Medicine (Jiangsu TCM 2018 No. 4).

## Conflict of Interest

The authors declare that the research was conducted in the absence of any commercial or financial relationships that could be construed as a potential conflict of interest.

## Publisher's Note

All claims expressed in this article are solely those of the authors and do not necessarily represent those of their affiliated organizations, or those of the publisher, the editors and the reviewers. Any product that may be evaluated in this article, or claim that may be made by its manufacturer, is not guaranteed or endorsed by the publisher.
